# A New Multiplex Genetic Detection Assay Method for the Rapid Semi-Quantitative Detection of Six Common Curable Sexually Transmitted Pathogens From the Genital Tract

**DOI:** 10.3389/fcimb.2021.704037

**Published:** 2021-08-23

**Authors:** Zhaoyang Sun, Jun Meng, Su Wang, Feng Yang, Tao liu, Xianping Zeng, Dijun Zhang, Haowei Zhu, Wenjing Chi, Yixin Liu, Wenrong Jiang, Li Ding, Yingxin Miao, Yong Wu, Hu Zhao, Yanmei Zhang

**Affiliations:** ^1^Department of Laboratory Medicine, Huadong Hospital, Affiliated With Fudan University, Shanghai, China; ^2^Key Laboratory of Clinical Geriatric Medicine, Huadong Hospital, Shanghai, China; ^3^Research Center on Aging and Medicine, Fudan University, Shanghai, China; ^4^Department of Laboratory Medicine, Ruijin Hospital, Shanghai Jiaotong University School of Medicine, Shanghai, China; ^5^Department of Research and Development, Ningbo HEALTH Gene Technologies Co., Ltd, Ningbo, China

**Keywords:** Sexually transmitted infections (STIs), High-throughput multiplex gene detection system (HMGS), rapid, semi-quantitative detection, pathogens, positive rates

## Abstract

**Background:**

Sexually transmitted infections (STIs) are some of the most common communicable conditions and exert impact on the health and lives of many hundreds of millions of people across the world every year. Screening high-risk populations and conducting comprehensive detection tests would lead to a significant improvement in preventing the transmission of STIs and help us to provide rapid treatment to those affected. Here, we successfully established and validated a novel high-throughput multiplex gene detection system (HMGS) for the simultaneous and semiquantitative detection of six important curable sexually transmitted pathogens in a single reaction from secretions samples.

**Method:**

Fluorescently labeled primers were designed to target specific conserved and single-copy gene fragments of Ureaplasma urealyticum (U. urealyticum), Mycoplasma hominis (*M. hominis*), Chlamydia trachomatis (*C. trachomatis*), Neisseria gonorrhoeae (*N. gonorrhoeae*), Trichomonas vaginalis (*T. vaginalis*), and Treponema pallidum (*T. pallidum*). The specificity and sensitivity of the STI-HMGS was validated and optimized using plasmids and quantitative genomic DNA. Next, we validated the performances of the STI-HMGS for clinical application by testing samples of clinical secretions collected from patients who visited the gynecology and urology outpatient clinics of our reproductive medicine center. Results derived from the STI-HMGS were then compared with three approved commercialized kits that used to detect *U. urealyticum, C. trachomatis* and *N. gonorrhoeae*, respectively, followed by further validation with Sanger sequencing for all pathogens. Finally, a comprehensive analysis of epidemiology was performed among different subgroups to investigate the association between infection rates and clinically-relevant information.

**Results:**

The sensitivity of STI-HMGS for six target genes was 10 copies/µL. Data derived from the detection of 381 clinical secretions demonstrated that the STI-HMGS exhibited high concordance rate compared with approved commercialized kits and almost 100% sensitivity and specificity for the detection of six sexually transmitted pathogens when validated by Sanger sequencing. Semi-quantitative analysis found that STIs caused by *N. gonorrhoeae* had a significantly higher (*P<0.05*) pathogen load than the other pathogens. Infections caused by *C. trachomatis* were significantly more common in younger individuals (*P<0.05*). We also found that *U. urealyticum* infections were more likely to happen in females; while the males were more affected by *N. gonorrhoeae* (P<0.05).

**Conclusions:**

STI-HMGS proved to be an efficient method for the semi-quantitative detection of six important curable sexually transmitted pathogens and therefore represents an alternative method for the clinical detection and monitoring of STIs.

## Introduction

Sexually transmitted infections (STIs) have profound effects on the sexual and reproductive health of the global population and represent one of the top five conditions for which adults seek health care. It has been reported that more than 1 million people contract a STI every day (Sanz-Lorente et al., 2018). The World Health Organization (WHO) has periodically produced estimates of the global and regional burden of four of the most common curable STIs: chlamydia (etiological agent: *C. trachomatis*), gonorrhoea (*N. gonorrhoeae*), trichomoniasis (*T. vaginalis*) and syphilis (*T. pallidum*) approximately every 5 years since 1995 (WHO, 2016). It is estimated that approximately 357 million people contract any of these four curable STIs each year (WHO, 2018).

CT and NG are the first and second most common forms of sexually transmitted bacterial pathogen worldwide, respectively (Martinelli et al., 2019). T. pallidum, an extracellular and highly motile bacterium, affects millions of people around the world every year; this bacterium is highly contagious and capable of infecting and damaging multiple tissues and organs (Bittencourt et al., 2015; Luo et al., 2018). T. vaginalis infection can lead to severe vaginitis and adverse pregnancy outcomes, including preterm delivery and low birth weight (Margarita et al., 2020). Numerous studies have proved that T. vaginalis can establish a symbiotic relationship with the M. hominis bacterium (Margarita et al., 2016).

*U. urealyticum* and *M. hominis* are both forms of genital mycoplasma and exist mainly in the genitourinary tract; these have both been implicated in genital and urinary infections (Pichon et al., 2019; Xiang and Lu, 2019). *U. urealyticum* is also a highly prevalent pathogen that is transmitted sexually across the world (Cai et al., 2019). *M. hominis* is the second smallest species, in terms of physical size, of self-replicating mycoplasmas and is commonly found in the upper genital tract (Henrich et al., 2017; Tsevat et al., 2017). Concern is increasing with regards to *U. urealyticum* and *M. hominis* as the causative factors responsible for non-gonococcal urethritis, cervicitis, pelvic inflammatory disease (PID), infertility, abortion, and ectopic pregnancy (Lopez-Arias et al., 2019; Klein et al., 2020).

These pathogens cannot be grown using normal microbiological culture methods, except *N. gonorrhoeae* which is fastidious and often will also be culture negative but PCR positive. Molecular diagnostic methods for gene detection technology, such as polymerase chain reaction (PCR) and high-throughput sequencing are useful for the identification of microorganisms that are difficult to culture due to their slow growth, or because of complex procedural issues, or the need specific transport protocols and culture media (Broitman et al., 1992). However, high-throughput sequencing is expensive and is not recommended for the routine screening of these pathogens. Real-time PCR has been widely used in clinical molecular laboratory, but it is low-throughput and only used to analyze no more than four target genes in a single tube (Li et al., 2019). At the present time, a comprehensive screening program for the simultaneous detection of these six pathogens is not yet available and reported. Consequently, in the present study, we aimed to establish a new high-throughput multiplex gene detection system, which we referred to as STI-HMGS, for the semiquantitative detection of these six curable sexually transmitted pathogens. Our assay successfully detected these six targets in samples of clinical secretions from the genitourinary tract within 4 hours. Our assay will therefore help to identify the underlying pathogens in STIs in patients in a timely manner, help to determine the clinical etiological agents for clinicians, and reduce healthcare costs by providing a more accurate and timely medical diagnoses.

## Material And Methods

### Specimen Collection and qRT-PCR Detection

Sample collection took place between June and December 2020. We acquired samples of vaginal or urinary secretions from patients and physical examination population who visited the gynecology and urology outpatient clinics in the reproductive medicine center in Huadong hospital, affiliated with Fudan University. These patients and physical examination population were routine detected the *U. urealyticum*, *C. trachomatis* or *N. gonorrhoeae* by using three corresponding commercial qRT-PCR kits (Shanghai Rendu Biotechnology Co., Ltd, Shanghai, China) according to the product instructions, which were performed on LightCycler^®^ 480 Real‐Time PCR Instrument (Roche, Mannheim, Germany). *T. vaginalis* was detected by microscopy examination and *T. pallidum* was screened by serological examination.

For female patients, the clinical samples were collected by wiping mucus from the cervical surface using a sterile cotton swab. For male patients, samples of urethral secretions were collected by inserting a sterile cotton swab 1–2 cm into the urinary meatus and rotating. Swabs were placed into specimen tubes that contained 1.5 mL of the preservation solution to await further processing, including the qRT-PCR detection and STI-HMGS assays. This study was approved by the Ethics Committee of Huadong Hospital, affiliated with Fudan University (Ethics Approval Number: 20190112).

### Primer Design

We designed eight pairs of fluorescently labeled primers that targeted specific conserved genomic fragments within six curable sexually transmitted pathogens and two control genes. We also selected single-copy genes from each pathogen for semi-quantitative analysis. The human internal DNA control gene (Human DNA), which exists in human histiocytic cells, and is located from RP11-320F15 on human chromosome 10 was used as the quality control for STI-HMGS. The other control was based on the complete sequence of an artificial pseudovirus particle; this served as a systematic internal control (IC). The systematic internal control (IC) for STI-HMGS was an artificial pseudovirus particle; this is a specific nucleic acid fragment wrapped in a viral coat protein and has been widely used in nucleic acid detection reagents, gene diagnosis reagents, and scientific research reagents, as a positive control. A number of sequences for each target were downloaded from the National Center of Biotechnology Information (NCBI) and analyzed using Vector NTI (Invitrogen, Carlsbad, USA) to determine the most highly conserved and single-copy gene targets for each pathogen. Next, we designed primers to amplify each of the highly conserved regions using DNASTAR software (DNASTAR Inc., Madison, WI, USA) and Premier 6.0 software (Premier Biosoft International, Palo Alto, CA, USA). All of the primers were designed and optimized according to specific principles. First, the primer sequences needed to be homogeneous and amplify products of 100 – 400 base pair (bp) with at least 3 bp differences between each product. The primers also needed to amplify products without the formation of dimers and avoid the generation of non-specific products. The targets genes, primer sequences and corresponding amplicon sizes, are given in **Table 1**.

**Table 1:** Target genes, primer sequences, and amplicon sizes, for STI-HMGS.

Targets	Sequences (5′→3′)	size (bp)	Concentration (nM)
*T. pallidum*	F: TTGGTGATGAGTTGATTATGCGT	124	400
	R: GTGTTTAAATTCATCCATCCGCTAAC		400
*T. vaginalis*	F: TTCAGCAGATGAGGTACAAGAA	143	400
	R: GTAGTTCTGTGAAGTTAGTTTCCAA		400
*C. trachomatis*	F: AGGGCTTCCTTACCCATAAACTTACGCA R: ATGCTGTGACTTGTTGTAGTGTGTGAA	189	200 200
*U. urealyticum*	F: TGCCATAGAAATGCACGAGATTACAA	257	200
	R: GTTGGTGTACCATTCCAATACCAGTT		200
*M. hominis*	F: CGCCCATAAGTGCCTTACCAAGTATA R: TCTGATACCGCAACCGCTATTGTATAC	181	200 200
*N. gonorrhoeae*	F: TATCGATGCGGACACCCAATACCTGC R: GTTTGAAATCTCCGTTGCCCATACCGG	206	200 200
Hum DNA IC	F: TGTCCGTGCCCCAAATTCCAG R: GTCTCGGTCAGGTCTCCCA F: TTGATGGCACAGTCGAGGCTG R: GTGGCCGCTTTTCTGGATTCAT	308 315	100 100 200 200

### Plasmid Construction

Plasmids were constructed for the six target genes and the two control genes. These were used in the STI-HMGS assay to quantify pathogen load and provide adequate quality control. The construction and transformation of eight plasmids were processed as follows: (1) Ligation: 2 µL of target-amplified gene fragment, 0.5 µL of PMD-18T vector (Tiangen Biotech Co., Ltd, Beijing, China) and 2.5 µL of solution I (Tiangen Biotech Co., Ltd, Beijing, China) were mixed and added to a 0.2 mL microcentrifuge tube which was then held at 16°C for at least 45min; (2) Transformation: 5 µL of ligation products were mixed with 1 mL of *Escherichia coli* (E. coli) DH5α competent cells (Tiangen Biotech Co., Ltd, Beijing, China) in a 1.5 mL microcentrifuge tube and then processed as follows: incubation on ice-bath for 30min; 42°C thermal shock for 90s and kept on an ice-bath for 2min. Next, 600 µL of LB broth, without ampicillin resistance, was added and the microcentrifuge tube was placed in a shaker at 37°C for 45min; (3) Inoculation: 200 µL of transformed cells were inoculated onto the plates at 37°C for 12h; (4) Identification: the transformed cells that contained cloned fragments of the target gene on the plate were identified by sequencing; (5) after identification, the transformed cells were expanded and cultured; (6) the plasmids were extracted using the Plasmid Mini kit (Omega, GA, USA) in accordance with the manufacturer’s protocol.

Plasmid concentration was quantified by a Qubit 2.0 Fluorometer and a Qubit dsDNA HS Assay kit (Thermo Fisher Scientific, USA) in accordance with the manufacturer’s instructions. The DNA copy number was calculated by the following formula: [(6.02×10^23^ copy number/mol) × plasmid concentration (g/mL)/L × (MW g/mol) = copies/mL (MW: average molecular weight). We prepared a gradient series of concentrations for each plasmid (target/control) and used this to validate the semi-quantitative performance of the STI-HMGS in terms of copy number.

### The Extraction of Nucleic Acid for STI-HMGS

Total DNA were extracted from 300µL of each clinical sample using the Smart LabAssist system and reagents (Taiwan Advanced Nanotech, Taiwan, China) in accordance with the manufacturer’s protocol. The IC was added to the extraction reagent prior to the extraction as an internal reference to assist with quality control. No any pretreatment such as centrifugation was required for STI-HMGS assay.

### Multiplex Polymerase Chain Reactions

Multiplex PCR included six components: 2µL of Roche Buffer, 0.5µL of uracil-DNA glycosylase (UNG), 0.4µL of FastStart *Taq* polymerase, 1µL of pooled primers, 1.1µL of ddH_2_O, and 5µL of template; the final volume of the PCR was 10µL. The primer pool consisted of six pairs of primers for the pathogens and two pairs of primers for the control genes. The primers were mixed in different proportions in order to achieve optimum sensitivity for all targets, the final concentration of each primer in the primer pool was listed in **Table 1**. The PCR mixture was incubated with the following cycle criteria by using the Veriti96-well Thermal Cycler (Applied Biosystems, California, USA): 42°C for 5 min; 94°C for 8 min; 34 cycles of 94°C for 30 seconds, 60°C for 30 seconds, 70°C for 1 min; and 72°C for 1 min.

### Separation by Capillary Electrophoresis and Fragment Analysis

Following the completion of the multiplex PCR, 1µL of the PCR product was added to 9µL of highly deionized (Hi-Di) formamide that contained 4% of DNA Size Standard 500 (Applied Biosystems, California, USA). Then, the PCR products were analyzed by the Applied Biosystems 3500DX genetic analysis system (Applied Biosystems, California, USA) based on the size separation caused by high-resolution capillary gel electrophoresis. Finally, the specific signal of PCR product was reported, the signal position indicated the amplified fragment length (bp) and the signal intensity was represented in the form of peak height and the peak area simultaneously. The results were considered to be positive when the peak height was > 500 relative fluorescence units (rfu). Throughout the entire detection process, we used ddH_2_O as a negative control.

### The Establishment and Optimization of the STI-HMGS Assay

The STI-HMGS assay was established and optimized using a series of specific principles: (1) primer sequences were optimized so that the signature of each detection target could be amplified specifically without interference; and (2) the system was systematically adapted and optimized with regards to the PCR components and procedures, including the concentrations and proportions of primers, PCR buffer, enzyme, reaction time, and the temperature of each step. The primers used to amplify the Hum DNA and IC were also included in the STI-HMGS PCR primer mix. The detection of Hum DNA in the clinical samples indicated that no significant nucleic acid degradation had occurred during specimen handling/storage. The IC (2.5×10^3^copies in 5µL) was added to the 300 µL extractions immediately prior to nucleic acid extraction and served as an internal control throughout the entire process of the STI-HMGS assay. The appearance of both internal control peaks in the STI-HMGS trace confirmed that the samples of DNA obtained from the clinical samples had good integrity, that the nucleic acids has been extracted efficiently, and that multiplex PCR amplifications and capillary electrophoresis had been carried out successfully.

### Specificity, Sensitivity, and Accuracy of the STI-HMGS Assay

The specificity of the STI-HMGS assay for the eight target genes was tested with the corresponding plasmids and validated by Sanger sequencing. Five negative control pathogens species, which all associated with the normal microflora of the human genital tract, were also used in the STI-HMGS assay to evaluate non-specific amplification, including *Escherichia coli*, *Enterococcus faecalis*, *Lactobacillus*, *Staphylococcus*, and *Gardnerella vaginalis* (Huang et al., 2019; Ma et al., 2019; Shridhar and Dhanashree, 2019). The eight plasmids were mixed in equal proportions to obtain a pooled plasmid mixture in which the eight DNAs were present in the same concentration. The sensitivity of the STI-HMGS assay for the eight target genes was tested by serial ten-fold dilutions of the corresponding plasmids. Then, the serial dilutions of the pooled plasmid solution, containing equal amounts of the eight templates, were used to test the simultaneous detection limit of the STI-HMGS for all target genes. To assess the accuracy of the STI-HMGS assay in polymicrobial mixtures, we selected different concentrations of two specific plasmids [*T. vaginalis* (10 copies/µL) and *T. pallidum* (10^1^ - 10^6^ copies/µL)] and mixed these for testing in the STI-HMGS assay; these results were then compared with the single-template STI-HMGS assay. In addition, the detection cost of STI-HMGS is $3 per sample, which was inexpensive and cost-effective.

### Determination the Limit of Detection (LOD) of STI-HMGS

Droplet Digital PCR (ddPCR) was used to the absolute quantification of genomic DNA of each pathogen from clinical specimens. The ddPCR copy number analysis was conducted on the Sniper DQ24 Digital PCR Platform (Sinper-tech, Suzhou, China). For each pathogen, the genomic DNA from clinical specimens were diluted to 10000 copies/µL, 1000 copies/µL, 100 copies/µL and 10 copies/µL, then detected by STI-HMGS. Following the guidelines in the Clinical Laboratory Standards Institute (CLSI) document EP17-A, the lowest concentration at which at least 19 of the 20 (95%) test results were positive was the LOD.

### Date Analysis and Statistics

The specificity (SP) and sensitivity (SE) of the STI-HMGS assay were calculated according to the following formulas: SE=TP/(TP+FN) ×100% and SP=TN/(TN+FP) ×100%; the positive predictive value (PPV) and negative predictive value (NPV) were calculated as follows: PPV=TP/P and NPV=TN/N (FN: false negative; FP: false positive; N: negative; P: positive; SE: Sensitivity; SP: specificity; TN: true negative; TP: true positive). The distribution of peak areas for the six pathogens were analyzed by GraphPad Prism version 8 software (San Diego, CA, USA). The Chi-squared test was used to compare the positive rates of infection among the genital tract infections group, infertile group, and the healthy controls. For all figures, *p < 0.05, **p < 0.01, ***p < 0.001, ****p < 0.0001, and ns (not significant*; p* > 0.05). A *p*-value < 0.05 was considered to be statistically significant.

## Results

### The STI-HMGS Assay Exhibited High Levels of Sensitivity and Specificity for All Target Genes

**Figure 1:**
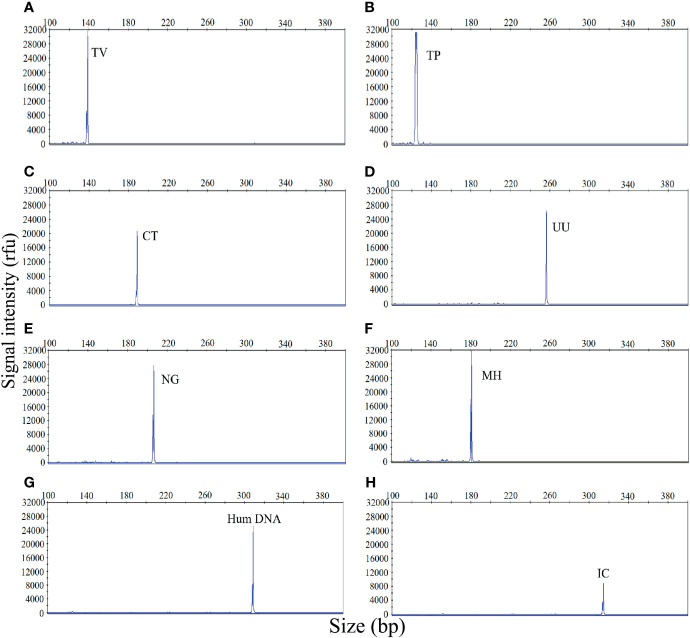
The sexually transmitted infection high-throughput multiplex gene detection system (STI-HMGS) assay produced specific amplification signals for 8 specific targets. The X-axis indicates the actual PCR product size while the Y-axis indicates the signal intensity. **(A-H)** Assay results arising from the amplification of 6 STI pathogens and two control genes: *Trichomonas vaginalis* (TV), *Treponema pallidum* (TP), *Chlamydia trachomatis* (CT), *Ureaplasma urealyticumm* (UU), *Neisseria gonorrhoeae* (NG), *Mycoplasma hominis* (MH), human internal DNA control gene (Human DNA) and systematic internal control (IC), respectively. All target genes were specifically amplified by the STI-HMGS assay without non-specific amplification.

The first goal of this study was to investigate the specificity of the STI-HMGS assay for all target genes. The specificity of the STI-HMGS assay for each pathogen and control gene was validated by using corresponding plasmids, which had been verified by Sanger sequencing. The assay produced specific amplification signals for all eight targets (**Figure 1**). Under the same experimental conditions, the DNA templates from five pathogens (for which specific primers were not included in our STI-HMGS assay) were also tested, including *Escherichia coli*, *Enterococcus faecalis*, *Lactobacillus*, *Staphylococcus*, and *Gardnerella vaginalis*. Results indicated that the STI-HMGS assay did not produce any specific amplification peaks from the five negative control pathogens. Furthermore, the STI-HMGS assay did not produce any positive detection signals from the ddH_2_O negative control (**Supplementary File 1** and **Figure S1**). For all pathogens and control genes, the corresponding plasmid for each target was serially diluted and detected individually by STI-HMGS. Results showed that the minimum detection limit was as low as 1 copy/µL for *T. pallidum* and 5 copies/µL for the other five pathogens (*U. urealyticum*, *M. hominis*, *C. trachomatis*, *N. gonorrhoeae* and *T. vaginalis*). Based on the absolute quantitation of the genomic DNA from ddPCR, the LOD of STI-HMGS for *T. pallidum was* 1 copies/µL and 10 copies/µL for the other five pathogens. STI-HMGS produced specific peaks that lay above the positive cut-of fluorescence signal of 500rfu when the nucleic acid concentration of pathogen was at or above the corresponding LOD. These results demonstrated that the STI-HMGS exhibited high levels of specificity and sensitivity for the pathogens and control genes tested herein.

### The STI-HMGS Assay Simultaneously Detected Six Pathogens in a Single PCR With High Levels of Sensitivity

**Figure 2:**
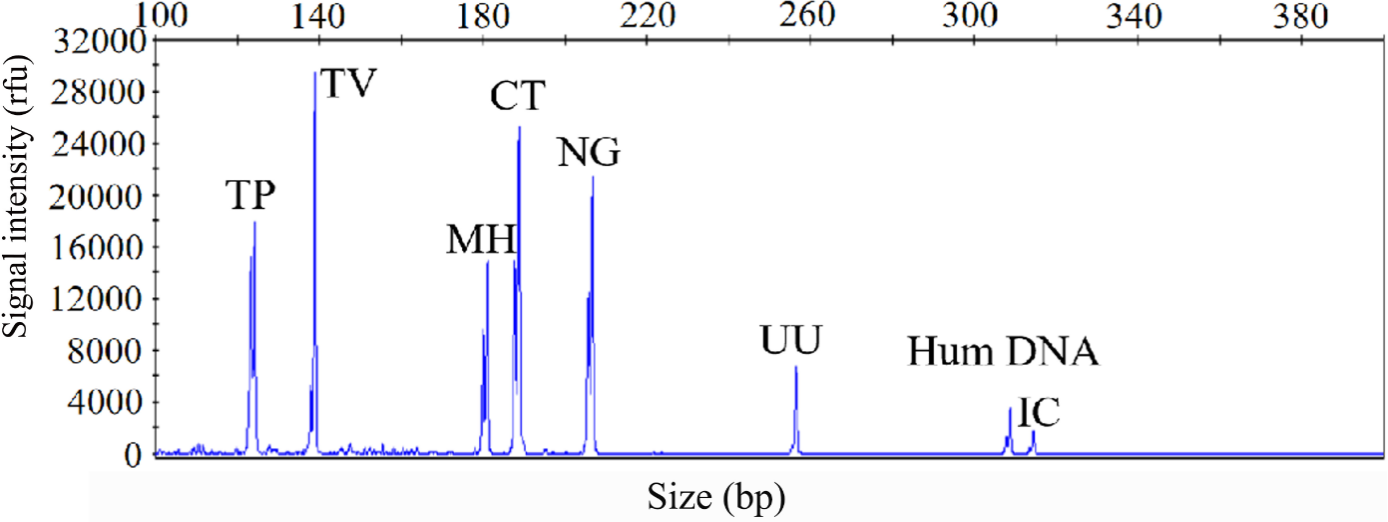
The optimized sexually transmitted infection high-throughput multiplex gene detection system(STI-HMGS) assay exhibited high levels of sensitivity for the simultaneous detection of all six pathogens and control genes in a single PCR. The detection limits of the STI-HMGS assay when detecting pathogens were determined by amplifying plasmids (diluted by ten-fold) containing equal concentrations of 6 pathogens and 2 quality control templates at a concentration of 100 copies/µL. The six pathogen-defining DNA targets all generated specific peaks, from left to right are *Treponema pallidum* (TP), *Trichomonas vaginalis* (TV), *Mycoplasma hominis* (MH), *Chlamydia trachomatis* (CT), *Neisseria gonorrhoeae* (NG) and *Ureaplasma urealyticumm* (UU). The human internal DNA control gene (Hum DNA) and systematic internal control (IC) produced specific peaks at 308bp and 315bp, respectively.

The sensitivity of the STI-HMGS assay was also validated for all targets using a gradient of concentration ranges of a mixture of the target plasmids at the same concentration. As shown in **Figure 2**, the assay was extremely reliable when detecting 100 copies/µL of the mixture of plasmids; this produced specific peaks that lay above the positive cut-off fluorescence signal of 500rfu for all targets. Thus, the sensitivity of the STI-HMGS assay for the simultaneous detection of all pathogens in a single reaction reached as low as 100 copies/µL. These results also indicated that the STI-HMGS assay can simultaneously detect six sexually transmitted pathogens in a single PCR.

### The STI-HMGS Assay Specifically Detected Individual Pathogens in Polymicrobial Mixtures

**Figure 3:**
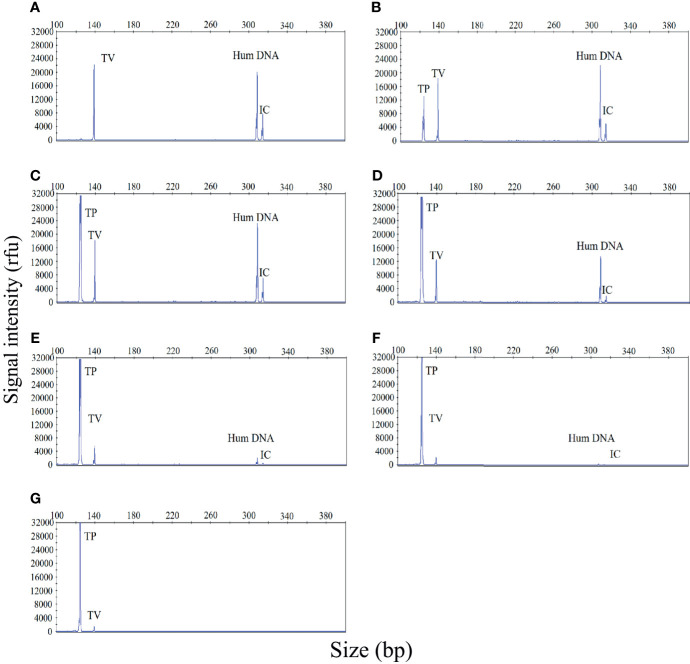
The sexually transmitted infection high-throughput multiplex gene detection system (STI-HMGS) assay accurately detected an individual pathogen in a mixture containing two DNA templates without interference from a dominant template over a large range of concentrations. **(A)** The *Trichomonas vaginalis* (TV) plasmid produced a signal intensity of 2200rfu. **(B–G)** Within the mixture of DNA templates, we increased the concentration of the *Treponema pallidum* (TP) plasmid from 10^1^, 10^2^, 10^3^, 10^4^, 10^5^ to 10^6^ copies/µL in a sequential manner while maintaining the concentration of the TV plasmid at 10 copies/µL throughout. The signal intensity for the TV plasmid showed progressive weakening from 20000, 18000, 12000, 6000, 2000, to 1000rfu. The two control genes were maintained at the same concentrations at all times; the signal intensity for these genes also showed a gradual reduction.

Polymicrobial infections are very common in cases of STI. Therefore, the ability to accurately identify multiple infections is essential if our STI-HMGS assay is to be applied effectively in the clinic. In the event of a polymicrobial infection, a mixture of different target templates, at different concentrations, may induce significant levels of competition within a single PCR, such that the signal produced by a template with a lower abundance could be attenuated by a template with a higher abundance, eventually leading to a false negative result. To demonstrate this hypothesis, a mixture of two pathogen-associated plasmids [*T. vaginalis* (10 copies/µL) and *T. pallidum* (10^1^ - 10^6^ copies/µL)] was analyzed by the STI-HMGS assay. As shown in **Figure 3**, when the *T. vaginalis* plasmid was analyzed individually by the STI-HMGS assay, the signal intensity of *T. vaginalis* at a concentration of 10 copies/µL was approximately 2200rfu (**Figure 3A**). Then, in a single PCR, we increased the concentration of the *T. pallidum* plasmid from 10^1^ - 10^6^ copies/µL while maintaining the concentration of the *T. vaginalis* plasmid at 10 copies/µL throughout. Under the gradually augmented interference caused by the *T. pallidum* plasmid, the signal intensity of the *T. vaginalis* plasmid progressively weakened from 22000rfu to 1000rfu (**Figures 3A–G**), but remained above the positive cut-off value of 500rfu. Thus, the STI-HMGS assay was able to accurately detect a DNA template at low abundance even in the presence of the strong impact from another detection target, consequently reducing the chances of a false negative result.

### Validation of the Semi-Quantitative Performance of the STI-HMGS Assay for Detecting Six Pathogens, as Determined by Copy Numbers

**Figure 4:**
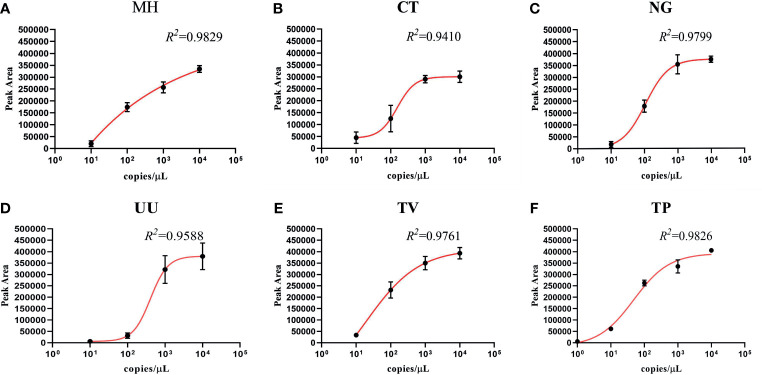
The detection of six pathogens from corresponding plasmids showing the quantitative standard curves obtained by 10-fold dilutions of the DNA template from 1 to 10^4^ copies/µL (x-axis) and their corresponding sexually transmitted infections high-throughput multiplex gene detection system (STI-HMGS) peak areas (y-axis). Each concentration was tested on three independent occasions using the STI-HMGS assay. Error bars are not shown if they are shorter than the size of the symbol used to indicate the mean value of the three peak areas. **(A–F)** Calibration curves for *Mycoplasma hominis* (MH) (R^2^ = 0.9829), *Chlamydia trachomatis* (CT) (R^2^ = 0.9410), *Neisseria gonorrhoeae* (NG) (R^2^ = 0.9799), *Ureaplasma urealyticumm* (UU) (R^2^ = 0.9588), *Trichomonas vaginalis* (TV) (R^2^ = 0.9761) and *Treponema pallidum* (TP) (R^2^ = 0.9826), respectively.

To investigate the semi-quantitative performance of the STI-HMGS assay and identify the pathogen load (copy number) in the nucleic acid extract from each clinical sample, we selected a single copy gene as the detection target. We then detected a range of plasmid concentrations (1 to 10^4^ copies/µL) on three independent occasions using the STI-HMGS. Then, we investigated the relationships between the different concentrations and the corresponding peak areas. As shown in **Figure 4**, the detection peak area obtained by STI-HMGS increased with increasing plasmid concentration for each pathogen; the R^2^ values for all six pathogens was >0.9000. This demonstrated that the STI-HMGS assay has the ability to accurately determine pathogen load in nucleic acid extractions from samples of clinical secretions. Theoretically, the concentration of a pathogenic template in a nucleic acid extract will increase with the actual pathogen content in the swabs used to collect secretions from the reproductive tract. In view of this, our STI-HMGS clearly represents potential and reliable method with which to semi-quantitatively detected six sexually transmitted pathogens in clinical samples.

### General Characteristics of the Subjects Recruited into This Study

In total, 381 individuals were recruited into the final cohort, including 143 patients showing the symptoms associated with genital tract infections, 124 patients who attended hospital due to infertility, and 114 healthy controls who received physical examinations and were devoid of any clinical symptoms. The clinical characteristics of the 381individuals recruited into this study are given in **Table 2**.

**Table 2:** The clinical characteristics of the 381 individuals recruited into this study.

Classifications	Number	Male/female	Age (years)
Based on recruitment reason:			
Genital tract infections	143	82/61	31.74 ± 7.543
Infertility	124	19/105	31.78 ± 7.596
Healthy controls	114	30/84	31.64 ± 7.665
Based on age distribution:			
11-20 years	7	2/5	19.00 ± 4.397
21-30 years	161	55/106	26.79 ± 2.644
31-40 years	173	60/113	34.38 ± 2.681
41-50 years	30	12/18	44.00 ± 2.857
51-60 years	10	2/8	53.00 ± 1.944
Total	381	131/250	32.13 ± 6.878

### The Detection of Pathogens From Clinical Samples by STI-HMGS Was Associated With Extremely High Levels of Accuracy Compared to qRT-PCR and Sanger Sequencing

After optimization, the STI-HMGS assay was used to detect clinical samples to evaluate its performance in practical applications. The STI-HMGS assay detected one or more pathogens in 123 (32.3%) of the 381 individuals tested; 258 (67.7%) were negative (signals from the two controls). The distribution of pathogens from the 123 patients who showed a positive detection result in the STI-HMGS assay are shown (in order of high-to-low proportion) in **Table 3**. *U. urealyticum* and *C. trachomatis* were both the most commonly detected pathogen and were identified in 35.0% of the 123 cases of STI; this was followed by *N. gonorrhoeae* (24.4%), *M. hominis* (22.8%) and *T. vaginalis* (2.4%), respectively. We did not detect *T. pallidum* infection in any of the samples tested.

**Table 3:** The distribution of pathogens detected in 123 patients by STI-HMGS.

Pathogens	Number of patients (n)	Percentage (n/123)
*U. urealyticum*	34	27.6%
*C. trachomatis*	31	25.2%
*N. gonorrhoeae*	23	18.7%
*M. hominis*	15	12.2%
*C. trachomatis* and *M. hominis*	4	3.3%
*M. hominis* and *U. urealyticum*	4	3.3%
*C. trachomatis* and *U. urealyticum*	2	1.6%
*M. hominis* and *N. gonorrhoeae*	2	1.6%
*C. trachomatis*, *N. gonorrhoeae* and *U. urealyticum*	2	1.6%
*C. trachomatis* and *N. gonorrhoeae*	2	1.6%
*T. vaginalis* and *U. urealyticum*	1	0.8%
*T. vaginalis* and *M. hominis*	1	0.8%
*C. trachomatis*, *N. gonorrhoeae* and *M. hominis*	1	0.8%
*C. trachomatis*, *M. hominis* and *T. vaginalis*	1	0.8%
Total	123	100%

In all enrolled 381 subjects, 106 were performed *U. urealyticum* detection by qRT-PCR, 130 were performed *N. gonorrhoeae* detection by qRT-PCR and 305 were performed *C. trachomatis* detection by qRT-PCR. The concordance rates of detection results between STI-HMGS and qRT-PCR were listed in **Table 4**. The overall concordance rates between them were 96.4%, 96.2% and 93.4% for the detection of *C. trachomatis*, *N. gonorrhoeae* and *U. urealyticum*, respectively. Note that there were 16 subjects were negative for qRT-PCR but positive for STI-HMGS, including eight *C. trachomatis*, four *N. gonorrhoeae* and *four U. urealyticum*.

**Table 4:** The concordance rates of detection results between STI-HMGS and qRT-PCR.

Pathogens	HMGS	qRT-PCR		The positive concordance rate (%)	The negative concordance rate (%)	The overall concordance rate (%)
		+	−			
*C. trachomatis*	**+**	25	8	89.3	97.1	96.4
	**−**	3	269			
*N. gonorrhoeae*	**+**	19	4	95.0	96.4	96.2
	**−**	1	106			
*U. urealyticum*	**+**	22	4	88.0	95.1	93.4
	**−**	3	77			

In addition, STI-HMGS detected *T. vaginalis* from one genital tract infection patient and two healthy controls, two of them were also found *T. vaginalis* by microscopic examination. All results from the STI-HMGS assay were further verified by Sanger sequencing, widely considered as the gold standard for gene identification (Baudhuin et al., 2015; Mu et al., 2016). The primer sequences, which were used to amplify the six pathogens by conventional PCR for Sanger sequencing are shown in **Supplementary File 2** and **Table S1**. Based on the results of Sanger sequencing, the detection accuracies for the STI-HMGS with regards to all pathogens were all extremely high across the 381 secretion samples (**Table 5**).

**Table 5:** STI-HMGS exhibited high levels of accuracy for the detection of six STI pathogens when compared to Sanger sequencing.

Pathogens	HMGS	Sequencing		Sensitivity	Specificity	PPV	NPV	Accuracy
		+	−					
*M. hominis*	**+**	28	**0**	1.000	1.000	1.000	1.000	1.000
	**−**	0	353					
*C. trachomatis*	**+**	43	1	1.000	0.997	0.977	1.000	0.997
	**−**	0	337					
*N.gonorrhoeae*	**+**	30	1	1.000	0.997	0.968	1.000	0.997
	**−**	0	350					
*U. urealyticum*	**+**	43	2	1.000	0.994	0.956	1.000	0.995
	**−**	0	336					
*T. vaginalis*	**+**	3	0	1.000	1.000	1.000	1.000	1.000
	**−**	0	378					
*T. pallidum*	**+**	0	0	/	1.000	/	1.000	1.000
	**−**	0	381					

### Comparisons of Positive Detection Rates Between the Genital Tract Infection Group, Infertility Group, and Healthy Control Group

**Figure 5:**
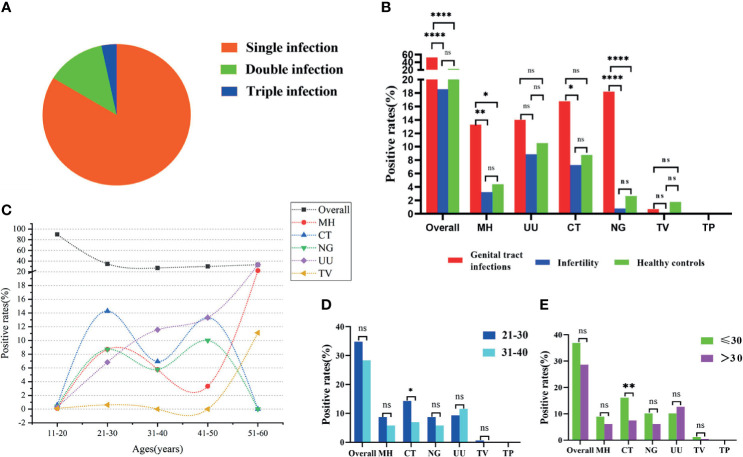
Analyses of clinical data based on Sexually transmitted infections high-throughput multiplex gene detection system (STI-HMGS). **(A)** STI-HMGS assays of the 123 confirmed STI patients showed that 83.7% of patients had a single infection, 13.0% had a double infection, and 3.3% had a triple infection. **(B)** A comparison of the positivity rates for each pathogen and the overall rate among the three groups. **(C)** The relationship between positive detection rates and different age groups. **(D)** A comparison of the infection rate between the 21-30 years group and the 31-40years group; only infections caused by *Chlamydia trachomatis* (CT) were significant. **(E)** Comparison of infection rate between the ≤30 years group and the >30 years group across the 381 subjects; only infections caused by CT were statistically significant. *p < 0.05, **p < 0.01, ****p < 0.0001, ns, not significant; p > 0.05.

The STI-HMGS assay revealed that 20 (16.3%) of the 123 patients with positive STI detection results were polymicrobial, including 16 (13.0%) cases of double infection and 4 (3.3%) cases of triple infection (**Figure 5A**). Patients with reproductive tract infections or infertility have a greater chance of developing STIs; this is why such patients were selected as our study population. One of the main aims of our study was to report and compare the infection rates of six sexually transmitted pathogens among the genital tract infection group, infertility group, and healthy control group. The positivity detection rates for each pathogen, along with the overall number of cases, were used to determine the proportion of positive patients; these data were then compared across the three groups. In this study, the ‘positive rate of overall’ refers to the proportion of patients that showed positive results by STI-HMGS, without considering the species and number of detected pathogens. The ‘positive rate of each pathogen’ refers to the proportion of patients who were detected with a specific pathogen. Results showed that the overall positive rates in patients with genital tract infections was significantly higher than patients who were infertile and healthy controls; the positive rates of *M. hominis*, *C. trachomatis* and *N. gonorrhoeae* in the patients with genital tract infections were all significantly higher (P<0.05) than the infertile patients; the positive rates of *M. hominis* and *N. gonorrhoeae* in the patients with genital tract infections were both significantly higher (P<0.05) than the healthy controls; the positive rates for the other pathogens were not significantly different when compared across the different groups (**Figure 5B**). Numerous previous have reported that curable STIs tend to occur more commonly in younger adults (Vieira-Baptista et al., 2017; Carnicer-Pont et al., 2019), thus providing motivation for the expanded analyses of the epidemiological distributions of STI frequencies across different age groups. We found that only the rates of *U. urealyticum* infection increased with increasing age; infections caused by *M. hominis*, *C. trachomatis*, *N. gonorrhoeae*, and *T. vaginalis*, all showed an increased tendency in the 21-30 years group (**Figure 5C**). The 21-30 years group (42.3%) and 31-40 years group (45.4%) showed similar proportions of infection caused by these pathogens, thus accounting for the highest proportion of infections within the 381 subjects. Consequently, we further compared the positive rates between these two groups. Results showed that the infection rate caused by *C. trachomatis* in the 21-30 years group was significantly higher than the 31-40 years group (**Figure 5D**); similar results were evident when comparing the ≤30 years group and the >30 years group when considering all 381 subjects (**Figure 5E**).

### The STI-HMGS Assay Identified a Significant Gender Difference for the Rate of Infections Caused by Some Pathogens

**Figure 6:**
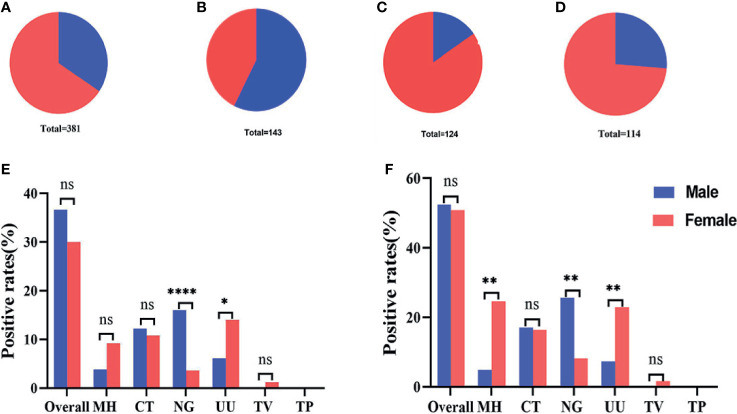
Gender ratio and infection rates across different groups. **(A–D)** Gender ratio across all 381 subjects, 143 patients with genital tract infections, 124 infertile patients, and 115 healthy controls. **(E)** The infection rates of 131 males compared with 250 females. **(F)** The infection rates of 82 males compared with 61 females in the group featuring 143 genital tract infections. *p < 0.05, **p < 0.01, ****p < 0.0001, ns, not significant; p > 0.05.

During the study period, our analyses showed that most of the 381 subjects recruited into this study were female, accounting for 65.6% (**Figure 6A**) of the final study cohort. Females also accounted for 84.7% (**Figure 6C**) and 73.7% (**Figure 6D**) of the group of infertile patients and healthy controls, respectively. A larger proportion of male patients (57.3%) were only observed in in the group of subjects with genital tract infections (**Figure 6B**). When considering all 381 recruited subjects, we found that infections caused by *N. gonorrhoeae* were significantly more common in males than females and that infections caused by *U. urealyticum* were significantly more common in females than males (P<0.05) (**Figure 6E**). Considering the gender ratio in the genital tract infection group was close to 1:1, we further analyzed the differences in infection rates within this group. Results showed that the positive detection rates for *M. hominis* and *U. urealyticum* in the females were both significantly higher (P<0.05) than for males; while the positive detection rate of *N. gonorrhoeae* detection in females was significantly lower (P<0.05) (**Figure 6F**).

### The STI-HMGS Assay Revealed a Higher Load of *N. gonorrhoeae* Infections in Clinical Samples

**Figure 7:**
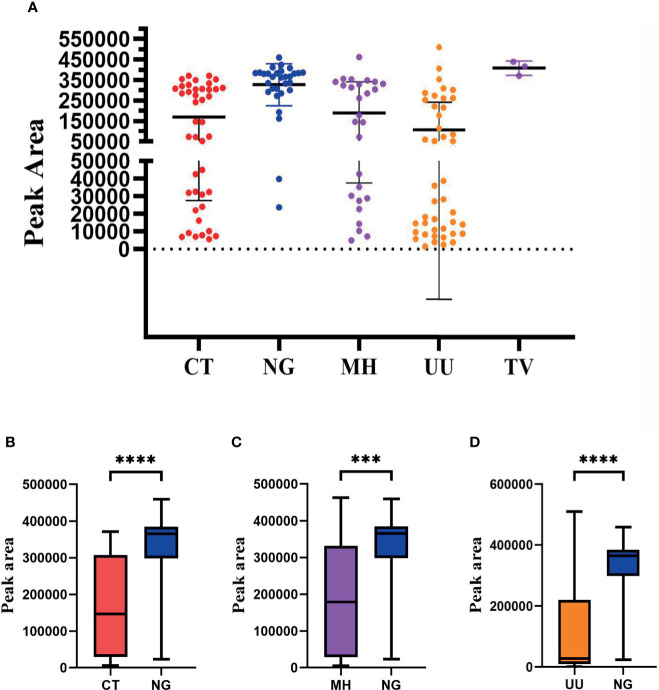
The distribution of peak area values for each pathogen as determined by the Sexually transmitted infections high-throughput multiplex gene detection system (STI-HMGS) assay from the 123 positive clinical samples compared to pathogen loading. **(A)** The peak area values for each individual pathogen, which showed a wide distribution between 0 and 550000, varied across the different clinical samples. **(B)** The level of the peak area values for *Neisseria gonorrhoeae* (*NG*) were significantly higher than the level of *Chlamydia trachomatis* (CT). **(C)** The level of the peak area values for NG was significantly higher than the level of *Mycoplasma hominis* (MH). **(D)** The level of peak area values for NG were significantly higher than the level for *Ureaplasma urealyticumm* (UU). ***p < 0.001, ****p < 0.0001; p > 0.05.

Our next goal was to carry out semi-quantitative analysis for all positive pathogens. Based on the performance of the semi-quantitative STI-HMGS assay, we found that the peak area produced by each individual pathogen varied across different samples (**Figure 7A**). According to the intuitive distribution of peak areas, the distribution of peak areas for *N. gonorrhoeae* were relatively concentrated at higher levels when compared with *C. trachomatis*, *M. hominis* and *U. urealyticum*, respectively. Then, we further compared differences in peak area between different groups. As shown in **Figures 7B–D**, the levels of the peak area all showed significant differences (*P*<0.05) when compared between *N. gonorrhoeae* and *C. trachomatis*, *N. gonorrhoeae* and *M. hominis*, and *N. gonorrhoeae* and *U. urealyticum*, respectively. Based on the semi-quantitative standard curve shown in **Figure 4**, the peak areas of these four pathogens corresponded to almost identical gene copy numbers; these could be used to represent the relative amount of each pathogen due to the fact that we selected single copy genes for all pathogens as detection targets.

## Discussion

*U. urealyticum*, *M. hominis*, *C. trachomatis*, *N. gonorrhoeae*, *T. vaginalis* and *T. pallidum* can all cause STIs and pose a serious threat to global human health. The traditional methods such as culture, qPCR and antigen detection techniques are available for the detection of these pathogens, but each method has its limitations in routine diagnosis for STIs. In addition, prior to this study, there was no established method that could simultaneously screen for these six pathogens in a single-tube PCR reaction by the semi-quantitative manner. Consequently, in this study, the successfully established new method, which enables the synchronized detection of these six STI pathogens from samples of clinical secretions and presents the advantage of rapid (less than 4h), high sensitivity (10 copies/uL), simple in operation (without any sample pretreatment) and inexpensive ($3 per sample), will allow comprehensive screen for STIs, especially in high-risk individuals and in many low-income countries. In addition, our new method will contribute positive effect on precision medication for STIs and curb the increase in drug resistance such as N. gonorrhoeae.

Following the establishment and optimization of the entire assay process, we found that the STI-HMGS assay could simultaneously and rapidly detect all six of these common sexually transmitted pathogens. Validation results for the STI-HMGS assay showed high levels of specificity for the target pathogens, without cross-reaction with other organisms. The LOD of the optimized STI-HMGS for six pathogens could all reached 10 copies/µL in term of genomic DNA of each pathogen; in other words, 50 copies per reaction. For *T. pallidum*, the LOD reached 5 copies per reaction.

In this study, some of detection results of STI-HMGS from 381 clinical samples were compared with three commercialized qRT-PCR, which are currently widely used in large numbers of Chinese hospitals and can only detect one of *U. urealyticum*, *C. trachomatis* and *N. gonorrhoeae* per test. Considering the suspected pathogen and detection price, not all patients will choose to test these three pathogens at the same time. Consequently, our new method may be a good alternative to qRT-PCR by virtue of the high concordance rate and the ability of comprehensive screening. Results from further Sangar sequencing showed 100% sensitivity and specificity for almost all pathogens, thus demonstrating that the STI-HMGS assay represented an extremely reliable tool for clinical application. By virtue of our multiplex PCR approach, our method revealed that 20 (16.3%) of the positive cases had multiple infection, thus highlighting the necessity for comprehensive screening strategies for STIs (**Figure 5A**). *M. hominis* was the most frequently co-infected organism (65.0%) and was present in 13 cases of multiple infection; this phenomenon was also reported in a previous study (Shim et al., 2010).

Within our study period, the overall positive rate for STIs was 32.3% in all 381 enrolled subjects. Patients with genital tract infection showed the highest overall positive rate (52.5%), followed by the healthy control group (22.81%) and the infertile group (18.6%) (**Figure 5B**). In a previous study, Imudia et al. found that almost one quarter of a group of infertile patients were positive for *U. urealyticum*, *M. hominis*, *C. trachomatis*, or *N. gonorrhoeae*, which are similar to our study (Imudia et al., 2008). Numerous previous studies shown that *C. trachomatis* is highly prevalent among the younger population (Huai et al., 2018; Martinelli et al., 2019); this conclusion is consistent with our present finding that infections caused by *C. trachomatis* were significantly more prevalent in the younger age group (*p*<0.05) (**Figures 5D** **, E**). We also carried out subgroup analysis based on different gender groups and found that infections caused by *M. hominis* and *U. urealyticum* were more significantly more common in females while infections caused by *N. gonorrhoeae* were significantly more common in males (*p*<0.05) (**Figure 6E**).

In order to semi-quantitatively detect six pathogens, we selected a single-copy gene for each target pathogen and limited the test volume of the preserved sample solution to 300 µL during the entire process of establishing and validating the STI-HMGS assay. Subsequently, we found a strong correlation (R^2^>0.900) between the various template concentrations and the corresponding detection peak areas when using a series of plasmid concentrations (**Figure 4**). This provided a solid basis for the semi-quantitative detection of pathogenic nucleic acids after extraction. On the basis of the distribution of peak area values from clinical samples the STI-HMGS assay showed that STIs caused by *N. gonorrhoeae* had a higher pathogenic load (**Figure 4**); this has not been reported previously. In addition, there were a significantly higher number of infections caused by *N. gonorrhoeae* in males than females (**Figures 6E, F**). Furthermore, cases of *N. gonorrhoeae* infection mainly occurred in patients with genital tract infection who exhibited relevant clinical symptoms (**Figure 5B**) (*P*<0.05).

One of the limitations of this study is that our new method was not compared with diagnostic gold standard for each pathogen. The reason for this lies in the diagnostic gold standards of six pathogens selected in our method have significant limitations and are unable to provide completely accurate results for STI diagnosis, including diagnosis or exclusion of infection in one hundred percent accuracy. For example, the diagnostic gold standard of *U. urealyticum*, *M. hominis*, *C. trachomatis* and *N. gonorrhoeae* was culture, but culture-based methods were constrained by low sensitivity and the negative result of culture does not completely rule out infection. This is also an important reason why there is an urgent need to develop the reliable new diagnostic methods to detect these STI pathogens and assist global public health efforts to control STIs.

## Conclusion

In this study, we successfully established a new multiplex assay (STI-HMGS) with extremely high levels of sensitivity and specificity for the semi-quantitative detection of six key STI pathogens from clinical samples. Preliminary application, based on 381 clinical samples, demonstrated that the new assay performed extremely well with clinical samples and revealed a correlation between the positive detection rates of various pathogens and subgroups. Collectively, the STI-HMGS assay exhibits great practicality and holds significant value for the routine surveillance, rapid diagnosis, and timely therapy, of patients with STIs.

## Data Availability Statement

The original contributions presented in the study are included in the article/**Supplementary Material**. Further inquiries can be directed to the corresponding authors.

## Ethics Statement

The studies involving human participants were reviewed and approved by the Ethics Committee of Huadong Hospital, Affiliated with Fudan University. Written informed consent to participate in this study was provided by the participants’ legal guardian/next of kin. Written informed consent was obtained from the individual(s), and minor(s)’ legal guardian/next of kin, for the publication of any potentially identifiable images or data included in this article.

## Author Contributions

Author Contributions has been revised to: ZS, JM, and SW contributed equally to this work. FY, TL, WC, YL, WJ, LD, YM, YW, HZ, and YZ contributed to the design and coordinated the study. ZS, JM, and SW collected the samples. XZ, DZ, and HWZ designed the primes. ZS performed the experiments,analyzed the data. ZS, JM, and SW wrote the manuscript. All authors contributed to the article and approved the submitted version.

## Funding

This work was supported by the Shanghai Science and Technology Committee (Grant No: 18411960600, 8411950800); National Natural Science Foundation Grant of China (Grant No.: 81602072); Shanghai Science and Technology Committee “Lead project” (Grant No.: 16411968000); Shanghai Shenkang Hospital Development Center “New frontier technology joint research project” (Grant No.: SHDC12015107); Ministry of Science and Technology “The National High Technology Research and Development Program of China (863 Program)”(Grant No: 2015AA021107-019); Shanghai “medical star” young medical talents training funding plan -outstanding young medical talents (HWJ HR [2019] No.72); “Huadong Hospital project” (Grant No: 2019jc020, 2019jc019).

## Conflict of Interest

Authors XZ, DZ, HWZ, and YW were employed by company Ningbo HEALTH Gene Technologies Co., Ltd.

The remaining authors declare that the research was conducted in the absence of any commercial or financial relationships that could be construed as a potential conflict of interest.

## Publisher’s Note

All claims expressed in this article are solely those of the authors and do not necessarily represent those of their affiliated organizations, or those of the publisher, the editors and the reviewers. Any product that may be evaluated in this article, or claim that may be made by its manufacturer, is not guaranteed or endorsed by the publisher.
