# Supplementary material for: A New Multiplex Genetic Detection Assay Method for the Rapid Semi-Quantitative Detection of Six Common Curable Sexually Transmitted Pathogens From the Genital Tract
Source: Front Cell Infect Microbiol. 2021 Aug 23;11:704037. doi: 10.3389/fcimb.2021.704037 (PMC8420868; doi:10.3389/fcimb.2021.704037)
Supplement: Supplementary file 1 [file Table_1.doc]

Supplementary Material

**Supplementary file 1: Figure S1**

The detection results of STI-HMGS for DNA templates from five pathogens that were not belonging to the target genes.


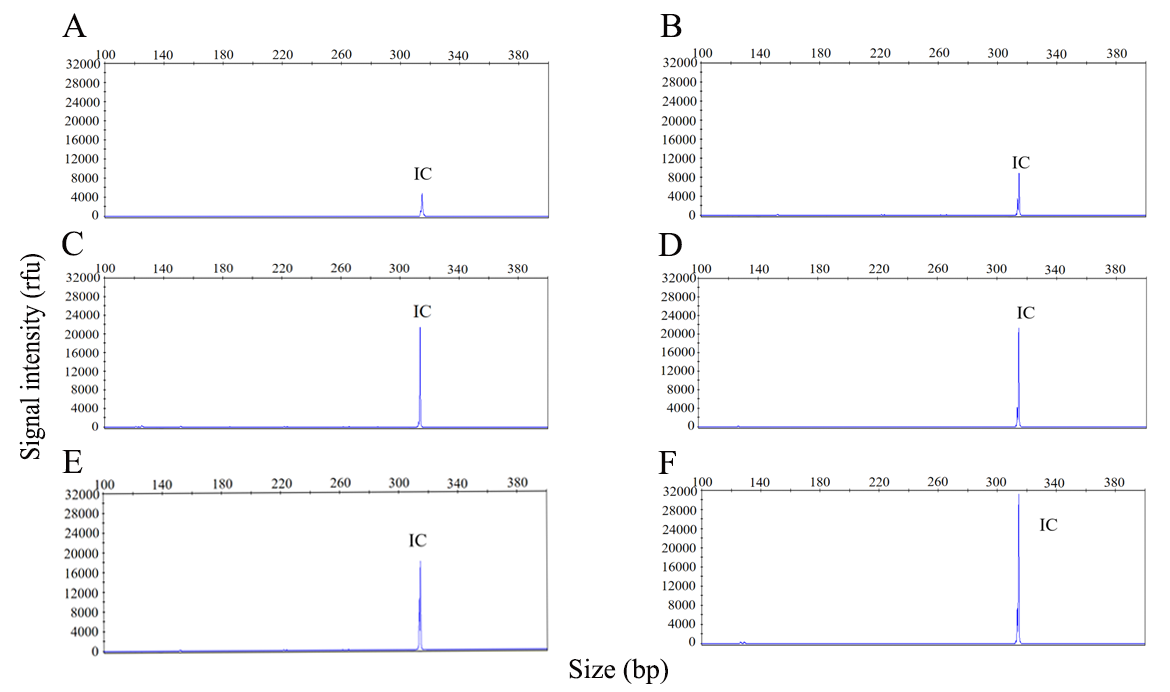


**Figure S1.** The STI-HMGS assay did not produce any non-specific amplification peaks for five negative control pathogens, same as the ddH2O. (A) The detection result of *Escherichia coli* (*E. coli*) by STI-HMGS. (B) The detection result of *Enterococcus faecalis* (*E. faecalis*) by STI-HMGS. (C) The detection result of *Lactobacillus*, *staphylococcus* by STI-HMGS. (D) The detection result of *staphylococcus* by STI-HMGS. (E) The detection result of Gardnerella vaginalis by STI-HMGS. (A) The detection result of ddH2O by STI-HMGS. All results above only produced the signal of IC.

**Supplementary file 2: Table S1**

The primer sequence of each pathogen for conventional PCR and sanger sequencing

| Target | sequences (5′→3′) |
| --- | --- |
| TP | F: TAATAAGTGTGCGTAAGCTGT |
|  | R: CATTTTCAAGATAGTAAATCGGCAT |
| TV | F: CTTGTTAAAAACGGTCTCGGAA |
|  | R: TCTTCTACTATGTCAGCCGAAT |
| CT | F: CGCAAATGGGCGGTAGGCGTGAGAAGACACAAACCCGCACA  R: GTTCACGGTGCCCTCCAAAGGGCCCAGGTTGATTGT |
| UU | F: CGCAAATGGGCGGTAGGCGTGAGATGCTGCTACAACTCCAGA  R: GTTCACGGTGCCCTCCTCATACCTAATTGTACTGTACCCTCA |
| MH | F: CGCAAATGGGCGGTAGGCGTGTTTATTGGGCTCGGTCACAG  R: GTTCACGGTGCCCTCCTGCATATCTTCAGAGTTACCTATTGTA |
| NG | F: CGCAAATGGGCGGTAGGCGTGAGCCGTACCTTCAACCTGATG  R: GTTCACGGTGCCCTCCGACCAAACCGATCAGAGAGGA |
